# The Influence of an Elevated Production of Extracellular Enveloped Virions of the Vaccinia Virus on Its Properties in Infected Mice

**DOI:** 10.32607/actanaturae.10972

**Published:** 2020

**Authors:** S. N. Shchelkunov, S. N. Yakubitskiy, T. V. Bauer, A. A. Sergeev, A. S. Kabanov, L. E. Bulichev, I. A. Yurganova, D. A. Odnoshevskiy, I. V. Kolosova, S. A. Pyankov, O. S. Taranov

**Affiliations:** State Research Center of Virology and Biotechnology VECTOR, Rospoterbnadzor, Novosibirsk region, Koltsovo, 630559 Russia

**Keywords:** smallpox, vaccine, immunogenicity, virulence

## Abstract

The modern approach to developing attenuated smallpox vaccines usually consists in targeted inactivation of vaccinia virus (VACV) virulence genes. In this work, we studied how an elevated production of extracellular enveloped virions (EEVs) and the route of mouse infection can influence the virulence and immunogenicity of VACV. The research subject was the LIVP strain, which is used in Russia for smallpox vaccination. Two point mutations causing an elevated production of EEVs compared with the parental LIVP strain were inserted into the sequence of the VACV *A34R *gene. The created mutant LIVP-A34R strain showed lower neurovirulence in an intracerebral injection test and elevated antibody production in the intradermal injection method. This VACV variant can be a promising platform for developing an attenuated, highly immunogenic vaccine against smallpox and other orthopoxvirus infections. It can also be used as a vector for designing live-attenuated recombinant polyvalent vaccines against various infectious diseases.

## INTRODUCTION

The vaccinia virus (VACV) belongs to the genus *Orthopoxvirus *of the family Poxviridae. This genus includes animal viruses such as the variola virus (VARV), the monkeypox virus (MPXV), the cowpox virus (CPXV), and others [1, 2]. Orthopoxviruses are the largest complexly organized DNA-containing mammalian viruses; their entire life cycle takes place in the cytoplasm of infected cells. The members of this genus are morphologically indistinguishable and antigenically closely related to each other. Therefore, infection with one species of orthopoxvirus provides protective immunity against other members of its genus [3]. For this very reason, the use of a live attenuated vaccine based on different VACV strains has made it possible to eradicate smallpox [1, 4].

Like other species of orthopoxviruses, VACV exists in two infectious forms. The virus progeny mostly consists of intracellular mature virions (IMVs) and a much smaller number of extracellular enveloped virions (EEVs) [5, 6]. IMVs accumulate in large amounts in an infected cell and are released into the environment only after the cell is destroyed. A small percentage of synthesized viral particles get enveloped with an additional lipoprotein coating and are released on the cell surface at the early stage of the viral replication cycle, where they are associated with the cell (cell-associated enveloped virions, CEVs). Some of these particles detach from the cells and exist in their free form (EEVs) [7]. EEVs make up less than 1% of all progeny of most VACV strains [5]. Meanwhile, the efficiency of EEV penetration into the cell is higher than that for IMVs [7, 8]; so, the virus quickly disseminates throughout the organism [5, 9]. No detailed studies of the effect of an elevated EEV production on the immunogenicity of VACV have been performed yet.

The VACV strains can differ substantially in terms of their level of EEV production [6, 10]. The IHD-J (International Health Department-J) strain is the most thoroughly studied variant of VACV that ensures a high yield of EEVs in the infected cell culture [6]. The *A34R *gene is one of the genes that regulate the release of CEVs to free EEVs [10]. Protein A34, contained in the lipoprotein envelope of EEVs, is not found in IMVs. The amino acid sequence of protein A34 of the neurovirulent mouse-adapted VACV Western Reserve (WR) strain ( < 1% EEVs among infectious virus progeny in the cell culture) differs from the amino acid sequence of this protein for the IHD-J strain (up to 30% EEVs) by only two point substitutions: Asp110 → Asn and Lys151 → Glu [10]. It was shown that replacement of the *A34R *gene in the VACV WR strain with the gene from the IHD-J strain significantly increases the yield of EEVs [9, 10].

It has been proved experimentally that the elevated EEV production caused by the insertion of mutations into the *A34R *gene leads to a more efficient dissemination of oncolytic variants of VACV and improves the *in vivo *antitumor activity of these viruses [9, 11]. However, the effect of these mutations on the virulence and immunogenicity of VACV has not been studied.

The cessation of smallpox vaccination after 1980 [2, 3, 4] has led to a situation where the contemporary human population is unprotected against the re-emerging orthopoxvirus infections [12]. Therefore, research focused on the development of novel, attenuated and highly immunogenic VACV-based vaccines becomes especially important [4].

VACV is extensively used not only to produce safe, next-generation live-attenuated vaccines against human orthopoxvirus infections, but also as a molecular vector in designing live recombinant polyvalent vaccines against various infectious diseases [3, 6, 12, 13, 14]. An important direction in research is the study of the effect of different viral genes and their mutant variants on the immunogenicity and safety of the vaccines being developed.

The objective of this study was to produce a LIVP VACV strain carrying mutations in the *A34R *gene that result in an elevated production of EEVs and to investigate the virulent and immunogenic properties of the LIVP-A34R variant compared to the parent LIVP strain when mice are infected via different routes.

## EXPERIMENTAL

**Virus, cell cultures**

In this study, we used the clonal variant 14 of the VACV LIVP strain (earlier described in [15]) and the African green monkey kidney cell cultures CV-1 and Vero from the cell culture collection of the State Research Center of Virology and Biotechnology (SRC VB) VECTOR, Rospotrebnadzor (Russian Federal Service for Surveillance, Consumer Rights Protection and Human Welfare). The viruses were grown and titrated on the CV-1 cell culture according to the procedure described in [16]. The Vero cell culture was used for the virus neutralization test conducted in the serum of mice.

**Production of VACV with point mutations in the** *A34R* **gene**

Two point mutations were inserted into the nucleotide sequence of the *A34R *gene by PCR with synthetic oligonucleotide primers; these mutations caused the synthesis of the protein corresponding to protein A34 of the IHD-J VACV strain (Asp110 → Asn and Lys151 → Glu substitutions) [17]. The recombinant LIVP-A34R strain carrying the mutant *A34R *gene was produced on the basis of the clonal variant 14 of the LIVP strain, using plasmid pMGCgpt-A34R* according to the procedure described previously [15].

**Animals**

Inbred BALB/c mice, both males and females, procured from the husbandry farm of SRC VB VECTOR, Rospotrebnadzor, were used in this study. The animals were fed a standard diet with a sufficient amount of water according to veterinary laws and regulations and in compliance with the National Research Council Guidelines on Laboratory Animal Care and Use [18]. All the manipulations on the animals were approved by the Bioethics Committee of the SRC VB VECTOR, Rospotrebnadzor.

**Assessment of the neurovirulence of VACV strains**

Suckling (2- to 3-day old) mice were challenged intracerebrally with the recombinant LIVP-A34R strain or the parent LIVP clonal variant diluted in normal saline (NS) at a dose of 10 pfu/10 µL/mouse. The animals in the control group received an identical volume of NS. The mice were followed up for 12 days; the number of animals that died was counted.

**Infecting mice**

The 3- to 5-week old BALB/c mice weighing 13–16 g were used. The animals were challenged with preparations of LIVP and LIVP-A34R viruses or normal saline intranasally (i.n.), subcutaneously (s.c.) or intradermally (i.d.) according to [16]. Infectious doses of 108, 107 or 106 pfu/30 L/animal were used. Each group consisted of 5–6 experimental animals. The mice were weighed daily, and external clinical signs of the disease (adynamia, tremor, and ruffled hair coat) were documented during 14 days.

**Collecting blood samples from the experimental animals**

Blood samples were collected from the retro-orbital venous sinus using sterile disposable capillaries on 28 dpi; then, the mice were euthanized by cervical dislocation. Serum was isolated from mouse blood by precipitating blood cells via centrifugation. Individual mouse serum samples were stored at 20°C.

**Enzyme-linked immunosorbent assay (ELISA)**

ELISA of individual mouse blood serum samples was performed according to [16]. A purified VACV LIVP preparation was used as an antigen. The geometric means of log reciprocal titer of VACV-specific IgG in experimental groups were calculated; the confidence intervals for a 95% matching between each sample and the total population were determined.

**Measuring the serum titers of virus neutralizing antibodies**

The titers of antibodies against VACV LIVP in mouse serum samples were quantified using the plaque reduction neutralization test (PRNT), according to the decrease in virus plaque count in a monolayer Vero cell culture, as described in [19]. Prior to performing PRNT, serum samples were inactivated at 56°C for 30 min. Four- to fivefold dilution series of serum samples, starting from a 1 : 10 dilution, in the cell maintenance medium were prepared. The dilution where 0.1 mL of the cell culture contained 30–60 pfu was used as the working dilution of VACV. The diluted serum samples and VACV solutions were mixed in equal volumes and incubated at 37.0 ± 0.5°C for 1 h. This mixture (0.2 mL) was placed onto the Vero cell monolayer in 24-well plates; 0.8 mL of the cell maintenance medium was added to each well, and the cells were cultured for 3 days in a CO_2_ incubator. After culturing, the monolayer was stained with a gentian violet solution and the plaque number in the wells was counted.

**Pathomorphological and virological analyses of the organs**

The mouse organs (lungs, brain, liver, kidneys, and spleen) and tissue samples (nasal septum or skin samples from the injection site) were collected from mice euthanized by cervical dislocation 3, 7, and 10 days post inoculation (dpi), with viral preparations or a normal saline solution. At each time point, organ and tissue samples from three animals were collected and analyzed individually.

To perform a postmortem analysis, mouse organs were fixed in a 4% paraformaldehyde solution (Sigma, USA) for 48 h. The samples were treated using the conventional procedure: sequential dehydration in alcohol solutions in increasing concentrations, impregnation in the xylene–paraffin mixture, and embedding into paraffin. Paraffin-embedded sections 4–5 m thick were prepared on a HM-360 automated rotary microtome (Germany). The sections were stained with hematoxylin and eosin. Optical microscopy studies and photomicrography were carried out on an AxioImager Z1 microscope (Carl Zeiss, Germany) using the AxioVision 4.8.2 software package (Carl Zeiss, Germany).

To perform the virological analysis, 10% homogenates of mouse organs and tissues were prepared by mechanical disintegration on a stainless-steel ball homogenizer, with a DMEM medium added subsequently. After several freeze–thaw cycles, the viral titers in the homogenates were determined on the CV-1 cell culture monolayer by viral plaque assay [15].

## RESULTS

**Production of the EEVs by LIVP and LIVP-A34R VACV strains**

Since VACV EEVs are released from the cell before the primarily infected cell is lysed and all the infectious viral forms get into the extracellular space, we conducted experiments where the viral titers in the infected cells and the extracellular fluid were quantified depending on the time post-infection. The CV-1 cell monolayer in a six-well plate was inoculated with LIVP or mutant LIVP-A34R produced from it with a multiplicity of 1 pfu/cell. Aliquots of the extracellular fluid were collected every 3 h for 1 day, and the cells contained in the growth medium were subjected to two freeze–thaw cycles. Viral titer in the samples was determined by viral plaque assay. Three replicates were recorded for each sampling point.

**Fig. 1:**
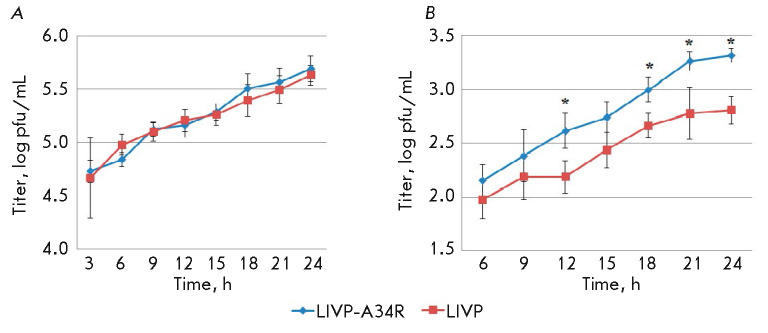
The dynamics of replication of different variants of VACV in the CV-1 cell culture. (*A*) – biosynthesis of the intracellular IMV form; (*B*) – accumulation of the EEV form in the extracellular medium. * – differences are statistically significant at *p *> 0.1

The results of these experiments (*Fig. 1*) demonstrate that the mutant LIVP-A34R does not differ from the parent LIVP in terms of the level of synthesis of the IMVs (*Fig. 1A*), while the EEVs are produced in a statistically significantly greater amount compared to LIVP (*Fig. 1B*). The exact percentage of the IMV and EEV forms in the total viral yield was not determined.

**The pathogenicity of VACV strains for different routes of inoculation of mice**

To perform a comparative analysis of the effect of the route of inoculation and the dose of the administered viral preparation on the pathogenic properties of the LIVP and LIVP-A34R strains, mice were infected via three of the most popular routes (the closest to the natural ones): intranasally (i.n.), intradermally (i.d.), or subcutaneously (s.c.). The infective doses of each virus were 106, 107 or 108 pfu/animal. Since inoculation of adult mice with most of the VACV strains usually does not cause animal death, the pathogenicity of the variants of this virus are studied according to the changes in body weight after inoculation and the clinical manifestations of the disease (ruffled hair coat, adynamia, and tremor) [20, 21].

**Fig. 2:**
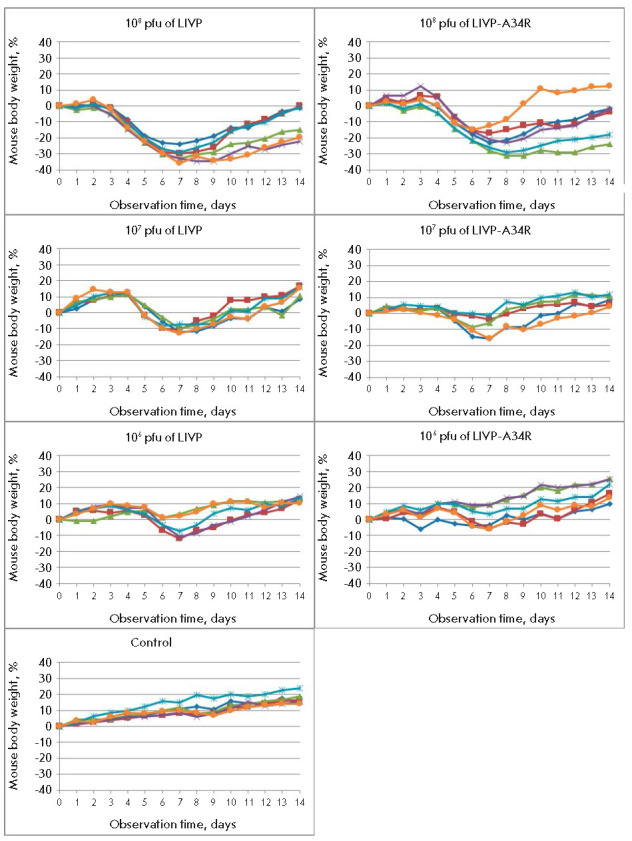
Changes in body weight of mice intranasally challenged with different doses of the LIVP or LIVP-A34R VACV strain or normal saline (control). The data for individual animals are presented

Marked clinical manifestations of infection and transient body weight loss were observed only after i.n. inoculation of mice with both VACV strains (*Fig. 2*). The peak of the disease occurred on 6–8 dpi. With increasing virus dose, clinical manifestations of infection became more pronounced and the decline in mouse body weight was more significant. *Figure 2* demonstrates that the mutant LIVP-A34R strain at doses of 106 and 107 pfu was characterized by the lowest pathogenicity.

**Fig. 3:**
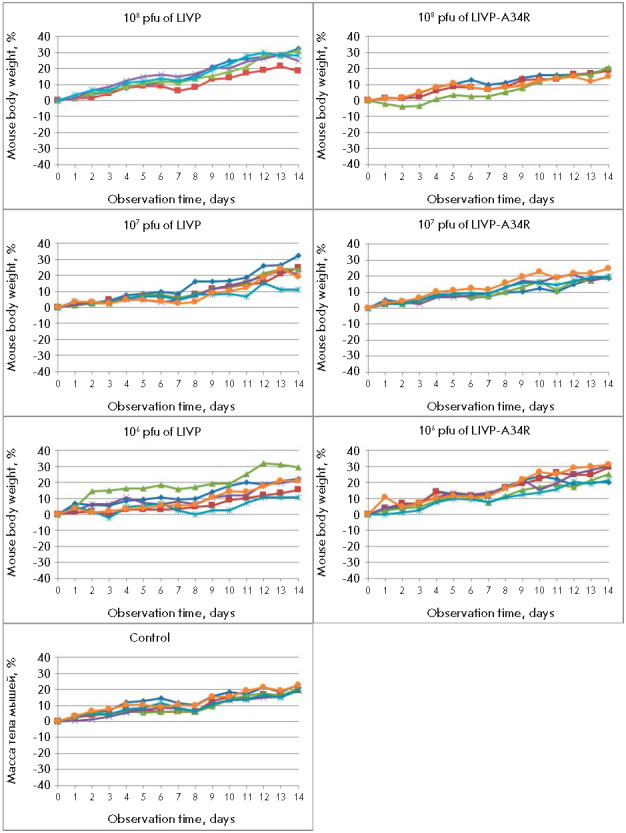
Changes in body weight of mice intradermally challenged with different doses of the LIVP or LIVP-A34R VACV strain or normal saline (control). The data for individual animals are presented

**Virus dissemination in the mouse organism**

The organ and tissue samples from animals i.n., i.d., or s.c. inoculated with the LIVP or LIVP-A34R strain at doses of 106, 107 or 108 pfu/animal collected on 3, 7, and 10 dpi were used to prepare 10% homogenates; viral titers were determined on the CV-1 cell culture monolayer by viral plaque assay. The lung, brain, liver, spleen, and kidney samples were analyzed. In i.n. inoculated mice, the nasal septum mucosa was additionally examined; skin flaps from the site of the virus inoculation were also analyzed in the animal groups that had received an i.d. or s.c. injection.

**Fig. 4:**
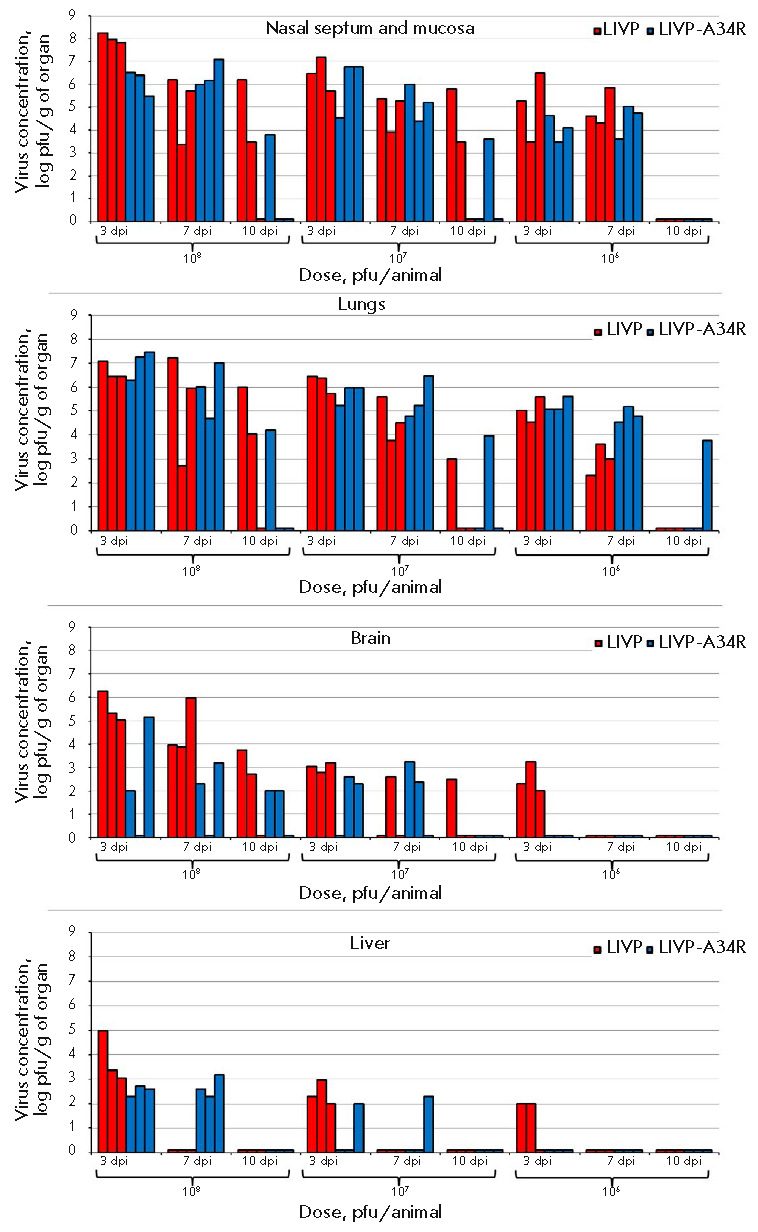
Accumulation of the VACV in organs and tissues in mice intranasally infected with different doses of the LIVP or LIVP-A34R strain. The data for individual animals are presented. dpi – days post-infection

In i.n. inoculated mice, the viruses were detected in all examined organs: the highest titers were revealed in the nasal septum mucosa (the primary virus replication focus), and in decreasing order, in the lungs, brain, liver (*Fig. 4*), kidneys, and spleen.

In animals s.c. inoculated with the analyzed VACV variants, the viruses were detected only in the skin flap samples collected from the site of injection of the viral suspensions at the maximal infective dose. No viruses were detected in the internal organ samples.

**Pathomorphological analysis of mouse organs**

In general, the pathological changes in the organs of experimental animal groups correspond to the histological pattern of the changes observed in the laboratory animals infected with orthopoxviruses [22], thus confirming the adequacy of the selected model. The severity and extension of the pathological changes varied depending on the virus strain, the infective dose, and the route of administration of the viral preparation.

The most typical pathomorphological manifestations of the infection were observed in the organs of the respiratory system, mostly in the lungs. The following manifestations were revealed in the respiratory tissue: profound swelling of the interalveolar septa, capillary hyperemia, and active release of blood cells and blood plasma into the alveolar space. In the most severe cases, exudation was accompanied by dystrophic and necrobiotic changes in the alveolar epithelium, fibrin accumulation, and mixed inflammatory cell infiltration (neutrophils, lymphocytes and a small amount of eosinophils). Macrophages were detected rarely, predominantly in mice i.n. inoculated with the virus at a dose of 108 pfu.

**Fig. 5:**
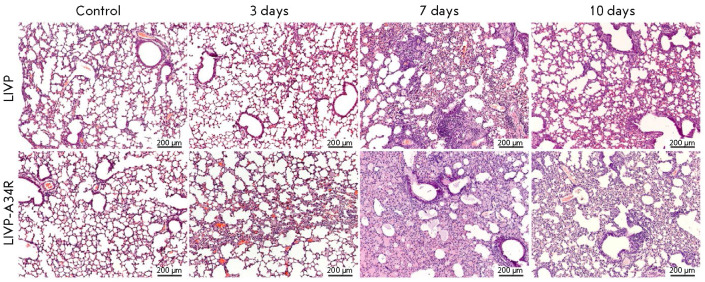
The dynamics of development of the pathological process in respiratory tissue after intranasal infection at a dose of 107 pfu (see explanation in the text). Histological lung specimen. Hematoxylin & eosin staining

In mice i.n. inoculated with the LIVP-A34R strain, reduced lung tissue airness, mild swelling, hyperemia, moderate infiltration of the stroma with lymphocytes and neutrophils were observed for the 1/5–1/4 of the section area on 3 dpi. In mice infected with the LIVP strain, changes in the lungs were minimal on 3 dpi (*Fig. 5*). The most pronounced pathomorphological signs of the disease were observed on 7 dpi: advanced severe swelling of interalveolar septa, polymorphic cellular infiltration, acute hyperemia and thrombosis of the microcirculatory vessels, and necrotic foci in the connective tissue surrounding major bronchi and blood vessels. Plasmorrhagia and fibrin exudation in the alveoli were more pronounced when mice were infected with the LIVP-A34R strain. The pathological manifestations were moderate on 10 dpi.

The pathological changes in the trachea and bronchi were mild, mostly manifesting themselves as sparse loci of epithelial dystrophy, thickening of the walls of small bronchi, moderate swelling of the intercellular spaces, and rarely, as epithelial desquamation, accompanied by the development of an erosive surface. Moderate peribronchial and perivascular polymorphic cellular infiltration was observed rather rarely and only in i.n. inoculated mice. The bronchi remained almost uninvolved in the pathological process in i.d. or s.c. inoculated mice.

**Neurovirulence of the VACV variants**

**Fig. 6:**
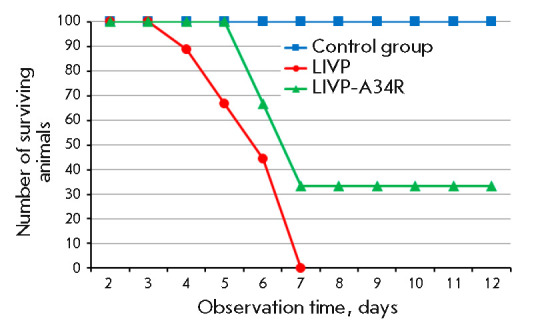
The death rate dynamics in newborn mice after intracerebral infection with the LIVP or LIVP-A34R VACV strain

The ability of the viruses to cause death upon intracerebral inoculation was studied in three groups of newborn mice (10 animals per group), which were followed during 12 dpi. In the group of animals inoculated with LIVP VACV at a dose of 10 pfu/mouse, the animals started to die on 4 dpi; all of them had died by 7 dpi. In the group of mice inoculated with the same dose of the mutant LIVP-A34R variant, the animals started to die on 6 dpi; after 7 pdi, no animal death was observed and a third of them survived (*Fig. 6*). No animal deaths were observed in the control group (mice that received an injection of NS).

**Immunogenicity of VACV strains**

The immunogenicity of the LIVP and LIVP-A34R VACV variants was assessed according to the titers of the virus-specific (ELISA) and virus-neutralizing antibodies (based on a reduction of the infectivity of the VACV preparation) induced by them in the mouse serum samples collected on 28 dpi via three different routes with different virus doses (106, 107 or 108 pfu/mouse).

A purified LIVP VACV preparation (the IMV form of the virus) was used as an antigen in ELISA tests. The results of ELISA (*Fig. 8*) demonstrate that, in i.n. inoculated mice, high titers of antibodies against the virion proteins of the IMV of VACV were detected for both the high (108 pfu) and lower infective doses. No statistically significant differences between the LIVP and LIVP-A34R strains were revealed for this parameter.

**Fig. 7:**
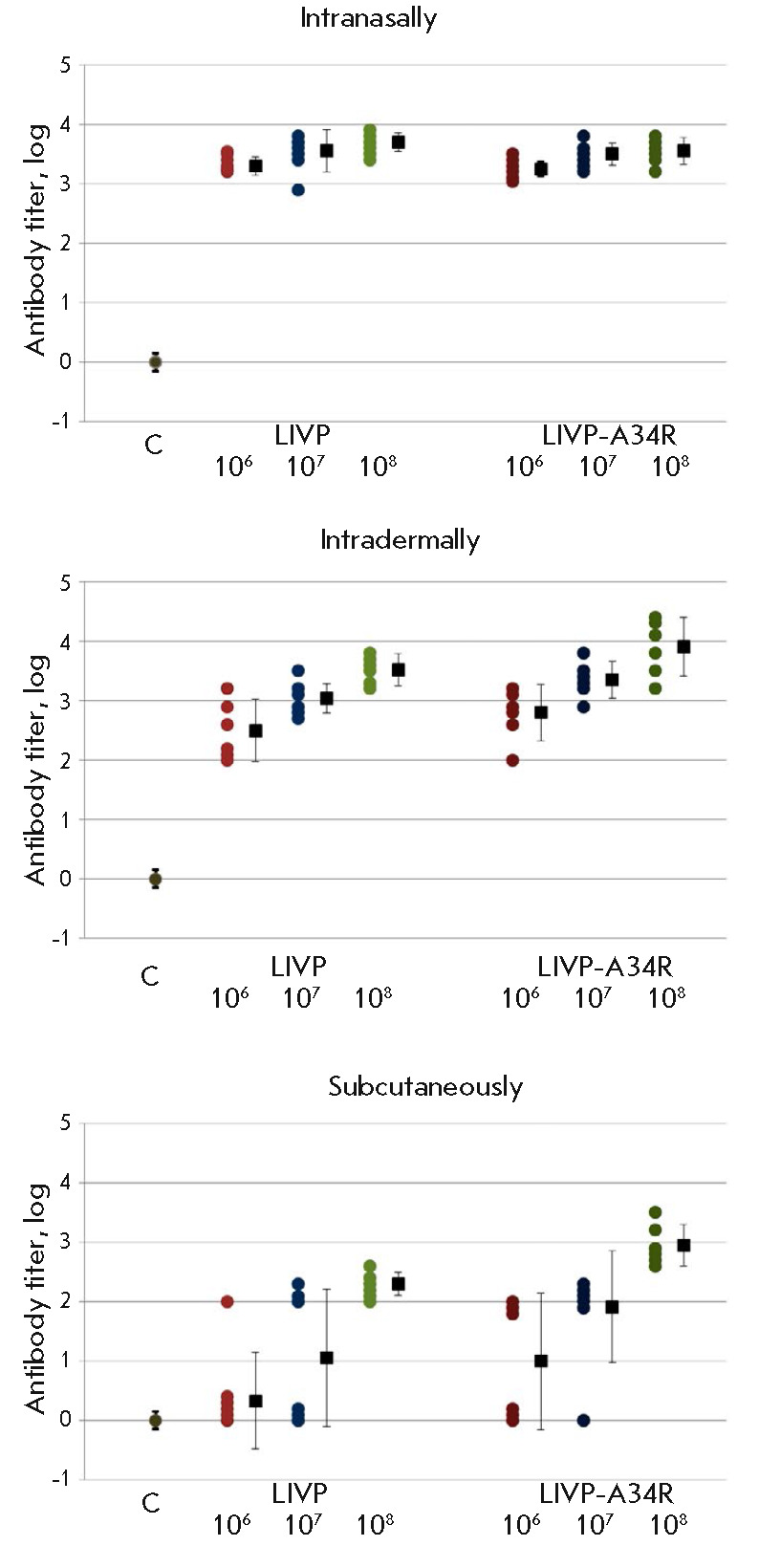
The titers of ELISA-determined VACV-specific antibodies in the serum samples of mice inoculated with the LIVP or LIVP-A34R strain at different doses through different routes. The data for individual animals and the geometric means of log reciprocal titer of VACV-specific IgG and confidence levels for the 95% matching between each sample and the total population are presented for each group

**Fig. 8:**
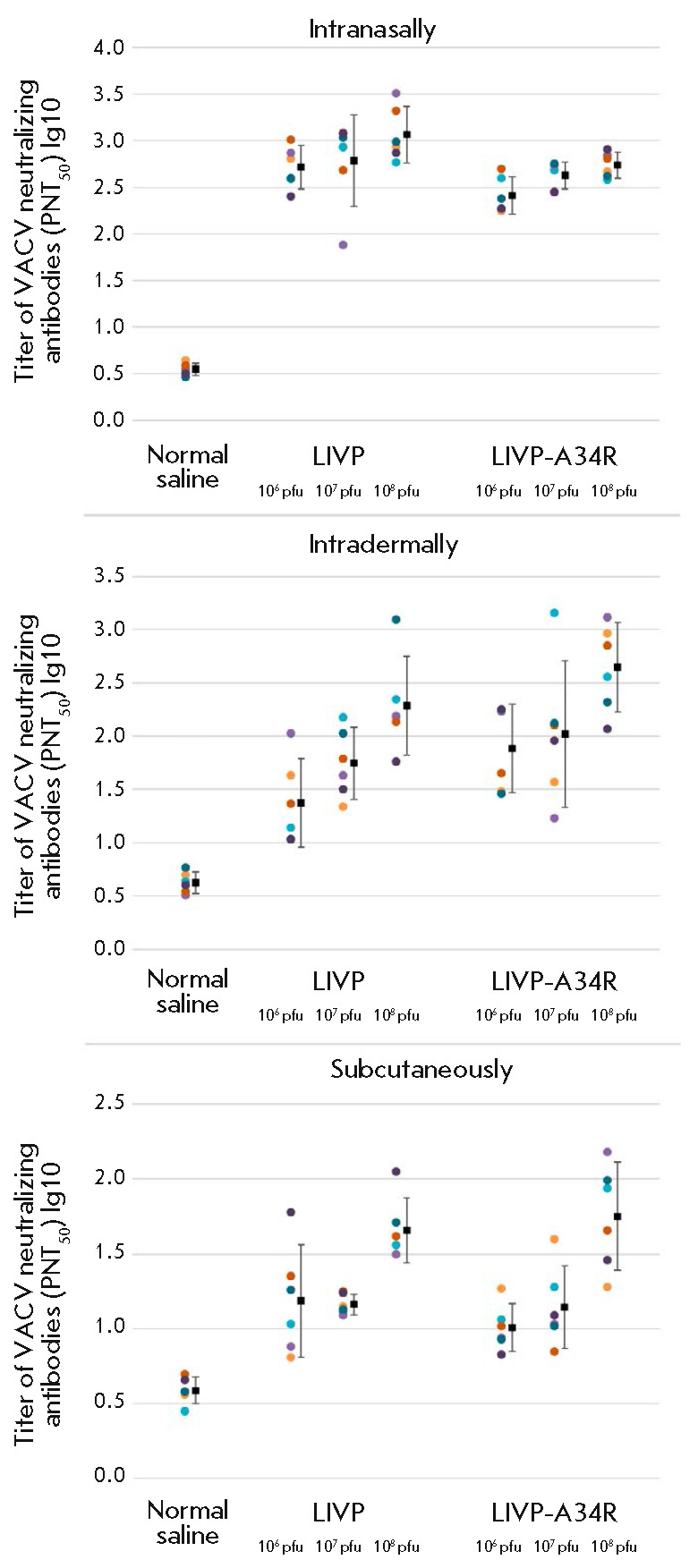
Levels of VACV-neutralizing activities of the serum samples collected on day 28 after the mice had been infected with LIVP or LIVP-A34R strains through different routes. The data for individual animals and the geometric means of log reciprocal antivirus neutralization titer and confidence levels for the 95% matching are presented for each group

The lowest titers of VACV-specific antibodies were detected in mice s.c. inoculated with the viruses. Antigen production was strongly dependent on the virus dose. The mutant LIVP-A34R strain ensured a higher production of virus-specific antibodies compared to the parent LIVP VACV strain (*Fig. 8*).

The titer of VACV-specific antibodies in mice i.d. inoculated with the virus at an infective dose of 108 pfu was comparable to the titer of antibodies elicited by i.n. inoculation with the viruses at the same dose. When the dose of i.d. inoculated virus decreased, biosynthesis of virus-specific antibodies fell more noticeably compared to the i.n. route of inoculation (*Fig. 8*). Meanwhile, the mean antibody titers were higher in the serum samples collected from mice inoculated with LIVP-A34R.

The findings obtained by analyzing the titer of virus neutralizing antibodies were similar to the ELISA data, but there were some differences (*Fig. 8*). The highest level of production of virus-neutralizing antibodies was detected in mice i.n. inoculated with the viruses. The LIVP strain produced higher levels of antibodies compared to the mutant LIVP-A34R variant. The weakest immune response was revealed in mice that received s.c. injections of the viruses. In i.d. inoculated mice, neutralizing antibodies were synthesized at a relatively high level, while the LIVP-A34R strain ensured a more efficient production of these antibodies compared to the LIVP strain.

## DISCUSSION

Vaccination was introduced over 200 years ago when ways to protect against smallpox were being developed. It remains the most reliable means for preventing viral infections [1, 6]. The conventional live smallpox vaccine was produced by replicating the VACV on the skin of calves or other domestic animals. This vaccine provided reliable protection against smallpox but in some cases caused severe post-vaccination adverse reactions (including encephalitis and encephalomyelitis, sometimes lethal) [1, 2]. Therefore, after the World Health Organization announced in May 1980 that smallpox had been eradicated, vaccination against this extremely dangerous human infectious disease was discontinued. As a result, today a large share of the world’s population has immunity neither against smallpox nor against other zoonotic orthopoxvirus infections such as the monkeypox, cowpox, camelpox, and vaccinia. The lack of herd immunity significantly facilitates the circulation of zoonotic orthopoxviruses in the human population [23-27]. Special concern is related to the human monkeypox, as its clinical presentation in humans is similar to that of smallpox, and the lethality of this infection can reach 10%. Furthermore, the efficiency of human-to-human transmission of the monkeypox virus has recently witnessed a manifold increase [23, 25].

In order to preclude a development of small outbreaks of orthopoxvirus infections into massive epidemics and reduce the risk associated with them due to the natural evolution of the orthopoxvirus, which is highly pathogenic for humans, researchers have focused their efforts on developing safe, next-generation live VACV-based vaccines [3, 6, 28].

The modern approach to designing attenuated, highly immunogenic vaccines usually involves a targeted inactivation of the VACV virulence genes [6, 15, 29, 30, 31]. Furthermore, the pathogenicity and immunogenicity of VACV depend on the virus strain and its route of inoculation into the animal organism [2, 6, 32, 33, 34, 35].

Earlier, it was shown using laboratory VACV strains that the VACV *A34R *gene is one of the key genes that regulate the detachment of the extracellular CEVs bound to the infected cell surface from the cell and their release into the environment (the so-called EEV form) [7, 10]. The *A34R *gene encodes a glycoprotein carrying a lectin-like domain within the outer membrane of VACV EEVs [7, 10, 36]. It turns out that protein A34 of the WR VACV strain producing less than 1% of virus progeny in the form of EEV in the cell culture differs from this same protein in the IHD-J strain (with the EEVs constituting up to 30–40% of its progeny) by only two amino acid residues at positions 110 and 151. Substitution of the *A34R *gene in the WR strain with a variant of this gene in the IHD-J strain significantly increases production of the EEV form, but the yield of EEVs typical of the VACV IHD-J strain is not attained [10]. Protein A34 performs its function (ensuring the release of EEVs from cells) by interacting with a number of other viral and cellular proteins [37] and provides efficient binding of EEVs to the cell and their penetration into the cell [8, 38].

It is believed that the C-terminal lectin-like domain of viral glycoprotein A34, which resides on the surface of extracellular virions (CEVs and EEVs) provides a highly specific interaction between this protein (and virions) and the carbohydrates on the cell surface [10]. The Lys151 → Glu substitution within this domain of protein A34 presumably reduces the efficiency of binding of the VACV virions released from the cell to the cell surface and increases the release of EEVs into the environment [10, 39].

The complex formed between the viral proteins B5 and A34 plays a crucial role in the binding of EEVs to the cell surface. The (80–130 a.a.) domain in protein A34 is the region where these proteins interact [38]. The Asp110 → Asn mutation in glycoprotein A34 of the IHD-J strain probably reduces the efficiency of complex formation between the proteins A34 and B5, thus decreasing the efficiency of CEV binding to the cell surface and additionally increasing the yield of EEVs.

We studied for the first time how an elevated production of the EEV form of VACV can affect the virulence and immunogenicity of the virus depending on its route of inoculation into laboratory mice.

The studies were conducted using the VACV LIVP strain, which is conventionally utilized for smallpox vaccination in Russia [2]. The clonal LIVP variant described earlier was used as the parent strain [15]. Two point mutations typical of the *A34R *gene in the IHD-J VACV strain were inserted. It was demonstrated for the CV-1 cell culture that the designed mutant LIVP-A34R variant produces a statistically significantly greater amount of EEVs compared to the parent LIVP strain (*Fig. 1*).

Intranasal inoculation of both VACV strains to BALB/c mice was shown to have a pathogenic effect, which was revealed through clinical signs and a reduction of mouse body weight (*Fig. 2*). Peak of the infection occurred on 6–8 dpi. The pathogenicity of the mutant LIVP-A34R VACV strain (assessed using these signs) was somewhat lower. Intradermal (*Fig. 3*) or subcutaneous inoculation of the viruses even at the maximal dose of 108 pfu neither reduced the mouse body weight nor led to the emergence of clinical signs of the disease.

An analysis of viral dissemination within the organisms of the experimental animals demonstrated that the highest *in vivo *viral dissemination was observed after i.n. inoculation; the viral load in the internal mouse organs depended on the infective dose (*Fig. 4*). It should be mentioned that more of the mutant VACV variant accumulated in the lungs of mice inoculated with the virus at doses of 107 and 106 pfu on 7 dpi (the peak of infection) compared to the parent LIVP strain.

Histological examination of mouse organs revealed the most typical manifestations of the infection in the organs of the respiratory system, mostly in the lungs. Pathological changes in the lungs in mice i.n. inoculated with LIVP-A34R appeared earlier than in mice infected with LIVP, being more severe because of the more significant involvement of microcirculatory vessels. Therefore, edema, plasmorrhagia and hemorrhagia developed to a greater extent on 3 dpi (*Fig. 5*). In both cases, the pathological manifestations decreased on 10 dpi.

Only the LIVP-A34R strain was detected in the liver in i.n. inoculated mice on 7 dpi (*Fig. 4*). All these findings demonstrate that LIVP-A34R is disseminated in the mouse organism more efficiently compared to LIVP. However, a lower level of LIVP-A34R accumulated in the brain compared to LIVP.

Intracerebral inoculation of the virus to newborn mice also demonstrated that the LIVP-A34R strain was characterized by reduced neurovirulence compared to the parent LIVP (*Fig. 6*).

For i.d. inoculated mice, the viruses were detected only in skin samples collected from the injection site of the viral preparation and at the maximum infective dose in the lungs and liver of some animals on 3 and 7 dpi. The amount of the mutant LIVP-A34R variant detected in the lungs was larger compared to that for the parent LIVP strain. No viruses were detected in the brain, spleen, or kidney samples.

In animals s.c. inoculated with the analyzed VACV variants, the viruses were detected only in the skin flap samples harvested from the injection site for the maximum infective dose. No viruses were detected in the internal organ samples.

It is known that the level of antibody response to vaccination against orthopoxvirus infections plays a decisive role in ensuring protection against a subsequent viral infection [6]. Therefore, we confined ourselves to studying the induction of biosynthesis of antiviral antibodies depending on the dose and route of inoculation of VACV into laboratory mice. The serum titers of the antiviral antibodies in mice inoculated with the viral strains under study were measured using two methods. The enzyme-linked immunosorbent assay was used to test the antibodies specifically interacting with the virion proteins of VACV IMVs. The titers of the antibodies that were bound to IMVs *in vitro*, thus suppressing their infectivity (plaque formation) upon the subsequent inoculation to the cell culture, were quantified in the same serum samples using the second method. Correlated results were obtained after i.d. inoculation of the viruses (*Fig. 8* and *Fig. 8*). Both viruses induced high production levels of both VACV-specific and VACV neutralizing antibodies; but LIVP-A34R elicited a stronger immune response.

The lowest level of production of antiviral antibodies was observed in mice s.c. inoculated with both LIVP and LIVP-A34R.

The maximum levels of production of both VACV-specific and virus – neutralizing antibodies were revealed in mice i.n. inoculated with the LIVP strain (*Fig. 8*, *Fig. 8*). For this route of inoculation, the mutant LIVP-A34R virus induced the formation of VACV-specific antibodies at concentrations comparable to those observed for the parent LIVP strain and a lower amount of VACV-neutralizing antibodies.

Hence, when inoculated through the i.n. route, both viruses exhibit pronounced pathogenicity, disseminate through internal organs, and therefore ensure a high level of induction of antiviral antibodies. The pathogenicity and neurovirulence of the LIVP-A34R strain are lower than those of LIVP. However, taking into account the high total virulence of the infection, this route of virus inoculation is hardly acceptable for the LIVP or LIVP-A34R strain being used as a live smallpox vaccine or a platform for designing a recombinant polyvalent vaccine

When inoculated s.c. to mice, both LIVP and LIVP-A34R exhibit low virulence and produce a low level of virus-specific antibodies.

route for inoculating both the parent LIVP strain and the mutant LIVP-A34R variant based on the pathogenicity/immunogenicity ratio. In addition to being detected in the skin at the injection site, after i.d. injection viruses are revealed by titration (the detection level, ≥ 102 pfu/g of the organ) on 3 and 7 dpi only in the lungs and liver of some animals solely at the maximum infection dose used (108 pfu). Meanwhile, a larger amount of the LIVP-A34R variant accumulated in the lungs compared to LIVP. The increased ability to disseminate in the organism could be the reason why the LIVP-A34R strain inoculated i.d. exhibited a higher immunogenicity compared to the parent LIVP variant.

## CONCLUSIONS

The results of this study demonstrate that, through the insertion of two point substitutions in the sequence of the A34 viral protein, the LIVP-A34R VACV strain produces more extracellular enveloped virions (EEVs) compared to the parent LIVP strain, is less neurovirulent, and induces an enhanced production of antiviral antibodies when administered intradermally. This variant of VACV can be used as a platform to develop a highly immunogenic, attenuated vaccine against smallpox and other re-emerging human orthopoxvirus infections. This variant of VACV can also be used as a molecular vector to design live recombinant polyvalent vaccines against various infectious diseases and oncolytic VACV variants.

## Abbreviations

pfu: plaque-forming unit
NS: normal saline
CEV: cell-associated enveloped virion
EEV: extracellular enveloped virion
IMV: intracellular mature virion
VACV: vaccinia virus
